# Improved HIV-1 Subtyping Accuracy Using near Full-Length Sequencing: A Comparison of Common Tools

**DOI:** 10.3390/ijms262311666

**Published:** 2025-12-02

**Authors:** Flavia Smoquina, Giulia Berno, Federica Forbici, Giuseppe Sberna, Gabriella Rozera, Isabella Abbate, Elisabetta Lazzari, Alessandra Amendola, Valentina Mazzotta, Roberta Gagliardini, Andrea Antinori, Enrico Girardi, Fabrizio Maggi, Lavinia Fabeni

**Affiliations:** 1 Laboratory of Virology and Laboratories of Biosafety, National Institute for Infectious Diseases Lazzaro Spallanzani-IRCCS, 00149 Rome, Italy; flavia.smoquina@inmi.it (F.S.); federica.forbici@inmi.it (F.F.); giuseppe.sberna@inmi.it (G.S.); gabriella.rozera@inmi.it (G.R.); isabella.abbate@inmi.it (I.A.); elisabetta.lazzari@inmi.it (E.L.); alessandra.amendola@inmi.it (A.A.); fabrizio.maggi@inmi.it (F.M.); lavinia.fabeni@inmi.it (L.F.); 2 Clinical and Research Department, National Institute for Infectious Diseases Lazzaro Spallanzani-IRCCS, 00149 Rome, Italy; valentina.mazzotta@inmi.it (V.M.); roberta.gagliardini@inmi.it (R.G.); andrea.antinori@inmi.it (A.A.); 3 Scientific Direction, National Institute for Infectious Diseases Lazzaro Spallanzani-IRCCS, 00149 Rome, Italy; enrico.girardi@inmi.it

**Keywords:** HIV-1, whole genome sequencing, near full-length, next generation sequencing, HIV-1 subtypes, circulating recombinant forms, molecular phylogeny, subtyping automated tools, genetic diversity

## Abstract

The extensive genetic diversity of HIV-1, also represented by the circulation of multiple subtypes and circulating recombinant forms (CRFs), poses significant challenges for accurate subtype classification, especially when sequencing is limited to partial genomic regions. This study evaluated the performance of four commonly used automated subtyping tools (Stanford HIVdb, COMET, REGA, and Geno2pheno) by comparing their outputs with molecular phylogenetic analysis (Mphy), considered the gold standard, using three NGS-derived sequence data sets: protease-reverse transcriptase (PR-RT), *pol*, and near full-length (NFL). One hundred plasma samples were processed to generate sequences of increasing length, which were analyzed to assess concordance, sensitivity, and specificity. NFL-based Mphy identified a higher proportion of circulating recombinant forms (51.6%) than PR-RT and *pol* (44.1%) and enabled the reclassification of 13 samples as more complex CRFs. Automated tools displayed good concordance with Mphy for PR-RT and *pol*, particularly for pure subtypes, whereas concordance decreased considerably for NFL sequences, especially among non-B subtypes and CRFs. Sensitivity varied substantially across tools and subtypes, while specificity remained consistently high. Overall, the findings indicate that whole genome or NFL sequencing enhances the detection of CRFs and that the accuracy of automated tools is strongly influenced by the completeness and updating of their reference databases.

## 1. Introduction

Human immunodeficiency virus type 1 (HIV-1) is characterized by remarkable genetic variability and rapid evolutionary dynamics. This diversity, driven by high mutation rate, recombination events, and global transmission, has led to the emergence of multiple subtypes and recombinant forms, particularly within group M, which is responsible for the global pandemic [1,2,3,4]. Group M is classified into ten pure subtypes (A, B, C, D, F, G, H, J, K, and L), of which A and F comprise numerous sub-subtypes (A1–A6, F1–F2). Additionally, genetic recombination between different subtypes simultaneously infecting a single individual gives rise to distinct viral variants, defined as unique recombinant forms (URFs) and circulating recombinant forms (CRFs) depending on whether they have been found in fewer than three epidemiologically unlinked individuals or in at least three such individuals, respectively. Currently, 167 CRFs are recorded in the Los Alamos National Laboratory (LANL) database (https://www.hiv.lanl.gov/components/sequence/HIV/crfdb/crfs.comp; accessed on 24 November 2025).

Molecular epidemiology studies have revealed a continuous evolution of the global geographical distribution of HIV-1 subtypes, mainly due to migration events and transmission within diverse populations. Historically, subtype B has been the most prevalent in Europe, especially in Western and Central regions. However, in recent decades, there has been a notable increase in the prevalence of non-B subtypes and newly emerging recombinants, which have shown an increasing prevalence in European countries [5,6,7]. Globally, pure subtypes A, B, and C account for approximately 70% of HIV-1 infections, whereas CRFs and URFs combined represent 29% [8].

The genetic complexity of HIV-1 has important implications for diagnosis, treatment response, disease progression, resistance mechanisms, vaccine development, and epidemiological surveillance [9,10,11,12].

For example, it was observed that subtype D is associated with a faster disease progression [13], while subtypes A and C have been found to have a longer asymptomatic period and slower progression to AIDS [11]. Regarding treatment response, sub-subtype A6, which has become increasingly prevalent in recent years [14], is characterized by the L74I mutation in the HIV-1 integrase gene as a naturally occurring polymorphism, which has been shown to represent a risk factor for treatment failure with long-acting injectable cabotegravir/rilpivirine [15,16].

Therefore, accurate identification of HIV-1 subtypes is crucial for supporting appropriate and targeted management of people living with HIV (PWH).

Usually, subtyping analysis is based on data derived from the HIV-1 genotypic resistance testing, which focuses on a specific region of the genome essential for assessing resistance to antiretroviral drugs: the *pol* gene, which encodes the protease (PR), reverse transcriptase (RT), and integrase (INT) enzymes. Although this region contains sufficient phylogenetic signal for confident subtype identification [17], this approach provides only a partial view of the viral genome and may limit the ability to detect complex recombinant forms by missing key recombination events or mutations in other regions, such as *gag* or *env*, which can influence viral fitness, immune escape, and transmission dynamics [18].

Whole genome sequencing (WGS) or near full-length sequencing (NFL) of HIV-1 can help overcome these limitations. By sequencing the entire viral genome, it is indeed possible to detect recombination breakpoints outside the *pol* gene and novel forms of the virus. This is particularly important in countries with high subtype diversity or where recombinant forms are prevalent. WGS enables high-resolution subtype classification, capturing complex recombination patterns that may be missed by partial genome analyses, also providing deeper insights into viral evolution and transmission dynamics. Moreover, this level of precision supports more valid and individualized treatment strategies and may ultimately contribute to improved therapeutic outcomes. In fact, the ability to detect and characterize CRFs/URFs is critical also for understanding transmission dynamics and guiding public health interventions [19].

Among other advantages of implementing WGS in HIV research and clinical practice, there is the ability to detect mutations across the entire genome, including in accessory genes and regulatory regions, that may influence drug susceptibility. This is particularly relevant for the detection of mutations for new classes of antiretrovirals targeting non-*pol* regions, such as the *gag*- and *env*-targeting antiretrovirals lenacapavir and fostemsavir.

Although the best method for accurate HIV-1 subtyping is molecular phylogenetic analysis (Mphy) of full-length genome sequences [1,20], this method is not usually integrated into routine clinical practice because of ongoing challenges related to the implementation of WGS or NFL, such as high cost, protocol management difficulties, and the complexity of data analysis. Recent advances, such as the adoption of next generation sequencing (NGS) amplicon-based strategies, technological advancements, and the fact that WGS protocols can be used by various sequencing platforms, are helping to make it more feasible and affordable, even in settings with limited resources.

Mphy is not widely implemented in clinical settings as well, due to its complexity and time-consuming nature of the procedures involved; therefore, most laboratories perform HIV-1 subtyping by using fast and easy-to-use automated tools available online, such as similarity-based tools like Stanford HIVdb [21] and Geno2pheno [22], statistical-based tools like COMET [23], or phylogenetic-based tools such as REGA [20].

In this study, we aimed at evaluating the performance of the above-mentioned automated tools in the classification of HIV-1 subtypes, assessing their concordance, sensitivity, and specificity with respect to the gold standard Mphy, using NGS-derived HIV-1 sequences of increasing length (*pol* gene region encoding PR-RT, entire *pol* gene, and NFL).

## 2. Results

### 2.1. Study Population

Samples from a total of 100 PWH were analyzed between 2024 and 2025. The population was mainly represented by male (79.4%), Italian (54.0%), and treatment-naïve (80.9%) subjects, with a median age of 39 years (interquartile range (IQR), 30 to 53 years). Overall, at the time of sample collection, median viremia level and median CD4+ cell count were 5.54 log_10_ copies/mL (IQR, 4.95 to 6.11 log_10_ copies/mL) and 204 cells/mm^3^ (IQR, 70 to 385 cells/mm^3^), respectively.

### 2.2. Performance of NFL Sequencing

The overall success rate of NFL sequencing was 93.0%. The seven samples for which sequencing was not optimal had a viremia level < 4000 copies/mL. NFL sequences had a mean (min–max) length of 8455 (5824–9578) nucleotides. The NFL sequencing success rate showed no statistically significant association with HIV-1 subtypes (*p* = 0.80).

### 2.3. Subtype Prevalence According to Mphy Based on PR-RT, pol, and NFL Sequences

**Figure 1:**
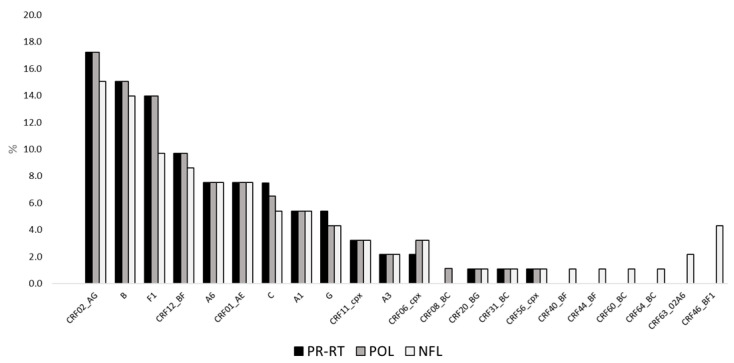
Subtype prevalence based on Mphy results performed on PR-RT, *pol*, and NFL HIV-1 sequences. Reference sequences of HIV-1 subtypes and CRFs were retrieved from the HIV sequence databases (https://www.hiv.lanl.gov/content/sequence/NEWALIGN/align.html, accessed on 1 September 2025).

Mphy analysis was conducted on PR-RT, *pol*, and NFL sequences separately, creating three different phylogenetic trees. Subtype prevalence according to Mphy assignments is shown in Figure 1.

By comparing the results, it was found that Mphy analysis based on NFL sequences allowed the characterization of a greater number of samples belonging to CRFs *(n* = 48, 51.6%) compared to Mphy based on PR-RT and *pol* sequences (both *n* = 41, 44.1%), allowing the identification of more complex recombinant forms. Moreover, 13 samples were reclassified with more complex subtypes as longer portions of the genome were considered, as shown in Table 1. Phylogenetic analyses of reclassified samples were shown in Supplementary Figure S1.

### 2.4. Concordance Between Automated Subtyping Tools and Mphy

**Figure 2:**
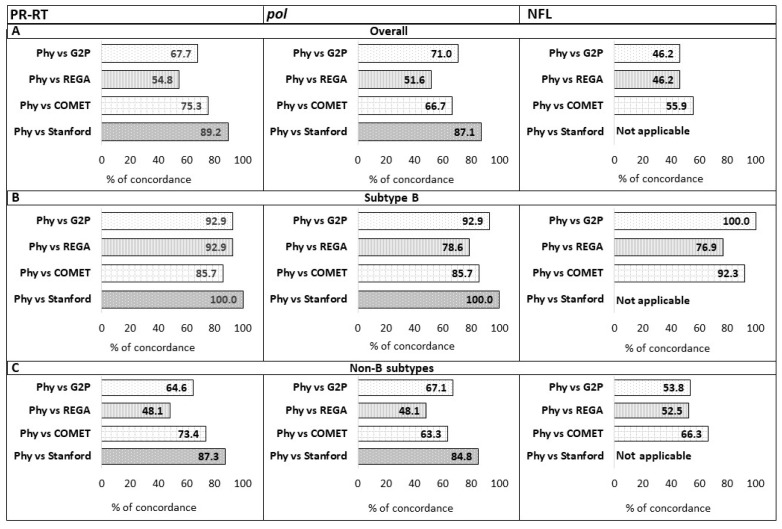
Concordance between HIV-1 automated subtyping tools (G2P, REGA, COMET, Stanford) and PR-RT, *pol*, and NFL Mphy in the overall population (**A**), in the subtype B population (**B**), and in the non-B subtype population (**C**). Not applicable: the concordance analysis between Stanford and NFL Mphy could not be carried out due to the lack of whole genome reference sequences in the tool database. Reference sequences of HIV-1 subtypes and CRFs were retrieved from the HIV sequence databases (https://www.hiv.lanl.gov/content/sequence/NEWALIGN/align.html, accessed on 1 September 2025).

With the aim of evaluating the performance of automated tools in terms of concordance, each HIV-1 subtype assigned by Mphy to PR-RT, *pol*, and NFL sequences was compared with those provided by Stanford HIVdb v9.8, COMET v2.4, REGA v3.46, and G2P v3.5. Discordance in subtype identification was defined as occurring when at least one subtyping tool output did not match the subtype assigned by Mphy. Samples were categorized into three groups: overall, subtype B, and non-B subtype populations (Figure 2).

In the overall population, the most concordant tool with PR-RT and *pol* Mphy was Stanford v9.8 (89.2% and 87.1%, respectively), while it was COMET with NFL Mphy (55.9%) (Figure 2A). Stanford was excluded in the NFL analysis because of the lack of whole genome reference sequences in its database, as described in the Materials and Methods section. When considering the assignment of subtype B (Figure 2B), concordance with PR-RT and *pol* Mphy was >85% for all the tools, except for REGA when using *pol* sequences (78.6%); considering NFL sequences, G2P was the most concordant tool (100.0%). When considering the assignment of non-B subtypes, concordance was generally lower, but Stanford remained the most concordant tool with PR-RT and *pol* Mphy (87.3% and 84.8%, respectively), while COMET reached the highest concordance with NFL sequences (66.3%) (Figure 2C).

### 2.5. Sensitivity and Specificity of Automated Subtyping Tools for Pure Subtype Assignment

Analysis of the performance of the four automated subtyping tools in terms of sensitivity and specificity with respect to Mphy results conducted on the three data sets is shown in Table 2. Only subtypes assigned to at least three sequences were included in the analysis. Overall, the most sensitive tool for both PR-RT and *pol* sequences was Stanford, which showed 100.0% sensitivity for all the subtypes, except for subtype G when using PR-RT sequences (80.0%). Regarding NFL sequences, each tool shows variable sensitivity to different subtypes. Considering subtypes B and G, the highest sensitivity was observed with G2P (100.0%), for subtype A1 with REGA (100.0%), for A6 with COMET (100.0%), and for F1 with REGA (88.9%). Considering subtype C, all the tools had a high sensitivity (100.0%). Of note, A6 was considered not applicable for REGA due to the absence of the reference sequences in the automated tool algorithm. The specificity of each tool was >90.9% for all three regions.

### 2.6. Sensitivity and Specificity of Automated Subtyping Tools for CRF Assignment

Overall, Table 2 shows that Stanford had the highest sensitivity (100.0%) in recognizing the considered CRFs for both PR-RT and *pol* sequences, except for CRF12_BF (55.6% for both PR-RT and *pol*). Considering NFL sequences, the highest sensitivity was shown by REGA and G2P for CRF01_AE (100%) and COMET for CRF02_AG (64.3%). Of note, because the G2P reference sequences list contains only pure subtypes, the tool was excluded from the comparison analysis with respect to CRF12_BF, since the “B, F1 recombinants” G2P output cannot be associated with any specific CRFs_BF. Nonetheless, CRF12_BF was not recognized by any tool when using NFL sequences. Regarding specificity, it was comparably high for all four tools (≥98.7%), but the most specific tool was COMET for all three data sets.

## 3. Discussion

In this study, we aimed at evaluating the performance of four commonly used automated subtyping tools (Stanford HIVdb v9.8, COMET v2.4, REGA v3.46, and Geno2pheno v3.5) in comparison with the gold standard Mphy, using three data sets of HIV-1 NGS sequences (PR-RT, *pol*, and NFL), in order to verify whether the accuracy of subtype assignment is dependent on the genomic region length analyzed. Consistent with previous work [24], which showed that the use of longer genomic regions improved the determination of subtype classification, particularly for CRFs, in our study it was shown that Mphy performed on NFL sequences provided the identification of a greater number of CRFs in comparison to using only PR-RT and *pol* sequences (NFL Mphy: 51.6% vs. PR-RT and *pol* Mphy: 44.1%). Moreover, the reclassification of thirteen samples into more complex CRFs by NFL Mphy shows how partial genome-based subtyping methods, although commonly used in clinical settings, may underestimate the true prevalence of recombinant strains. This underestimation can bias surveillance data and hinder the detection of emerging circulating forms. It also underscores that complex CRFs are not only challenging in terms of annotation but also in terms of recombination complexity, which is determined by the number of breakpoints [25,26]. Regarding automated tools, our results showed that the highest concordance with Mphy was observed with Stanford when PR-RT and *pol* sequences were analyzed, considering both subtype B (PR-RT and *pol*: 100.0%) and non-B subtypes (PR-RT: 87.3%, *pol*: 84.8%). Considering NFL sequences, G2P showed a better agreement with Mphy when subtype B was considered (100.0%), whereas it was COMET with non-B subtypes (66.3%). Of note, Stanford was not considered in the NFL analysis because of its lack of whole genome sequences in its references list, which highlights the importance of also updating tools’ databases in terms of sequence lengths. However, the overall concordance for NFL was generally lower across all tools (≥46.2%), suggesting that automated approaches present difficulties in accurately classifying HIV-1 subtypes, especially CRFs, again due to the absence of extensive and updated CRF reference sequences [27]. The only exception was CRF01_AE, for which all tools produced concordant results, probably because of its well-characterized structure and epidemiological importance in Asia [28,29,30]. The importance of regular database updating includes pure subtypes as well. The case of A6 represents a typical example: this sub-subtype, which is becoming increasingly prevalent in Eastern Europe and beyond [14,31], carries the L74I polymorphism in the integrase gene, a naturally occurring variant associated with reduced efficacy of long-acting regimens such as cabotegravir/rilpivirine [32,33,34]. Failure to correctly identify A6, either because of incomplete reference lists or the use of partial genome sequences, may therefore lead to clinically significant implications, directly affecting treatment outcomes. In this regard, samples of our data sets belonging to sub-subtype A6 based on Mphy are present in the overall PR-RT, *pol*, and NFL sequences with a prevalence of 7.5% and were correctly recognized by all the tools except for REGA, which wrongly classified it as A1. In addition, NFL Mphy characterized two more samples as CRF63_02A6, not recognized by any of the tools. Regarding this CRF, originally identified in 2006 in Siberia among people who injected drugs [35,36], it was described that surveillance drug resistance mutations were observed significantly more often in males and in PWH infected with this CRF compared to other variants [37]. Considering that A6 and its recombinants are not yet highly prevalent in other European regions but could spread given the critical situation of the ongoing war in Russia and Ukraine, it is of fundamental importance that subtyping tools’ reference databases are regularly updated, also including emerging subtypes, in order to ensure that automated tools remain accurate and clinically relevant in the context of a rapidly evolving epidemic. The comparison of automated HIV-1 subtyping tools using PR-RT, *pol*, and NFL sequences provided much information on their performance and revealed some limitations. Overall, our analysis confirmed that the accuracy of subtype assignment is not uniform but depends on the sequenced region analyzed, the subtype or CRF considered, and the tool employed. Considering pure subtype classification, Stanford consistently showed the highest sensitivity when using PR-RT and *pol* sequences, reaching 100.0% for most subtypes, with the only exception being subtype G in PR-RT sequences. When NFL sequences were taken into consideration, great variability in performance across tools and subtypes was observed. While G2P reached optimal sensitivity for subtypes B and G, REGA was better for A1 and F1, while COMET for A6. These findings once more confirm that the employment of automated tools presents significant challenges related to the fact that their reference databases are limited and may not be equally optimized for all subtypes. Of note, all tools achieved 100.0% sensitivity for subtype C, underlining that some major subtypes are more reliably detected regardless of the tool used. Regarding CRF assignment, our results show a more heterogeneous scenario. Stanford achieved the highest sensitivity for most CRFs in PR-RT and *pol* sequences but was less efficient in recognizing CRF12_BF. When using NFL sequences, performance varied substantially across tools, with REGA and G2P correctly identifying CRF01_AE and COMET showing the best sensitivity for CRF02_AG. In contrast, CRF12_BF was not detected by any automated tool. These results suggest that some CRFs, such as CRF01_AE, are generally well recognized due to their epidemiological importance and robust representation in reference databases, while others remain underdetected or misclassified. Specificity remained consistently high (≥90.9%) across tools and sequenced regions, indicating that false positives are a minor concern compared with the greater risk of underclassification or misclassification of specific subtypes/CRFs. Among the tools, overall COMET demonstrated the highest specificity. There are some limitations observed with this study. First, the number of analyzed samples, although sufficient to highlight subtype diversity, does not fully consider the global variability of HIV-1. Moreover, the underrepresentativeness of some non-B subtypes requires further investigation. Finally, only the most used automated tools were included in the present analysis, thus missing possible revisions of other existing tools. Altogether, our findings emphasize that, while automated tools are a user-friendly resource for rapid HIV-1 subtyping, their accuracy is highly context-dependent, therefore it is recommended to combine the use of more than one tool to overcome their limitations. Partial genomic regions (PR-RT, *pol*) are generally sufficient for reliable classification of major pure subtypes, particularly subtype B, but not for non-B subtypes and CRFs. By contrast, WGS or NFL sequencing enables the identification of complex recombination patterns and ensures more accurate classification of emerging CRFs and URFs, but at the cost of increased variability among automated tool outputs, which reflects the limited representation of CRFs in current reference tool databases. In conclusion, our findings highlight that WGS represents a crucial advancement in HIV-1 molecular epidemiology and clinical management, supporting its integration with Mphy as the gold standard for subtype determination [24,38,39], since it represents a powerful tool able to provide a comprehensive view of viral diversity, a reliable classification of complex variants that are underestimated by partial genome approaches, and the evaluation of the actual prevalence of HIV-1 recombinants [40,41,42]. This higher resolution is essential not only for robust epidemiological surveillance but also for clinical implications, such as resistance-associated variants outside the *pol* region and the spread of subtypes with specific therapeutic relevance, such as A6 and its recombinants. At the same time, continuous improvement and updating of automated subtyping tools, including the integration of complete genome reference data sets, and the simplification of WGS protocols, even in resource-limited setting laboratories, will be necessary to optimize both clinical practice and epidemiological surveillance with the advantages of WGS.

## 4. Materials and Methods

### 4.1. Study Population and Sample Collection

One hundred plasma samples were selected from HIV-1 routine genotypic resistance test samples processed in the National Institute for Infectious Diseases Lazzaro Spallanzani, IRCCS, Rome, Italy, considering the following inclusion criteria: viremia level > 400 copies/mL and subtype diversity. Viral RNA was extracted from 1 mL of plasma, following ultracentrifugation, using the QIAamp Viral RNA Mini Kit (QIAGEN, Hilden, Germany, 52904), according to the manufacturer’s protocol, and processed by NGS technology, as described in the next subsections, in order to obtain NFL sequences. All sequences were then collected in an anonymous database. Three different data sets of HIV-1 sequences were obtained: the PR-RT data set, containing the full-length PR and RT nucleotide regions (nucleotides 2253–3869 in the HXB2 genome); the *pol* data set, containing the full-length PR, RT, Rnase H, and INT regions (nucleotides 2253–5096 in the HXB2 genome); and the NFL data set (approximately from nucleotide 1 to nucleotide 9719 in the HXB2 genome).

### 4.2. HIV-1 pol Next Generation Sequencing

All samples were initially sequenced by NGS technology on the Ion GeneStudio S5 prime System platform (Thermo Fisher, Waltham, MA, USA) using an Ion AmpliSeq technology-based panel (Thermo Fisher) for the *pol* resistance region during HIV-1 routine genotypic resistance testing. Briefly, viral RNA was first reverse transcribed using the NGS RT kit (Thermo Fisher). Two amplicon pools were then obtained using 3 μL of cDNA each, in a final volume of 10 μL per pool, with the Ion AmpliSeq Library Kit 2.0 (Thermo Fisher). The resulting libraries were combined and digested with 2 μL of FuPa reagent included in the kit. Unique IonCode Barcode Adapters (Thermo Fisher) were ligated to each sample’s amplicon library. Library purification was performed using 40 μL of Ampure XP magnetic beads (Beckman, Brea, CA, USA), followed by two washes in 70% ethanol solution and elution in low TE buffer. DNA libraries were quantified with the Qubit dsDNA HS Assay Kit (Thermo Fisher), and concentrations were normalized to 50 pM. Samples were pooled in equimolar amounts. Template preparation of the 25 μL pooled samples and chip loading were automated using the Ion Chef instrument (Thermo Fisher), employing Ion 510/520/530 Chef Kits. Sequencing was performed on the Ion GeneStudio S5 prime System platform (Thermo Fisher). Raw sequencing data were then processed using Torrent Suite™ Software (version 5.18), including base calling, adapter trimming, and quality filtering. Reads were aligned to the HXB2 reference genome (GenBank accession number K03455) using Burrows-Wheeler Aligner version 0.7.13 (BWA-SW algorithm) [43], and variants were called using the Variant Caller plugin. Consensus sequences were generated using a minimum coverage threshold of 100 reads, as previously described [44].

### 4.3. HIV-1 NFL Next Generation Sequencing

All plasma samples were also processed for HIV-1 NFL sequencing. Particularly, the sequencing was performed on the Ion GeneStudio S5 prime System platform (Thermo Fisher) by using the Ion AmpliSeq WGS HIV-1 custom panel (Thermo Fisher; covering nucleotides 1–9719) to obtain NFL sequences encoding the *gag*, *pol*, *env*, *vif*, *vpr*, *tat*, *rev*, *nef*, and *vpu* genes. The NGS whole genome custom panel was constructed in collaboration with Thermo Fisher by using 184 full-length reference sequences of HIV-1 subtypes and CRFs retrieved from the HIV sequence databases, available at https://www.hiv.lanl.gov/content/sequence/NEWALIGN/align.html, accessed on 5 August 2025. Briefly, cDNA synthesis was first performed using the NGS RT kit (Thermo Fisher) with specific primers spanning the HIV-1 genome. NFL genome amplification was then achieved through PCR reactions targeting *gag*, *pol*, *env*, and accessory genes, yielding products that covered the approximately 9.7 kb HIV-1 genome. The protocol followed was as described above.

### 4.4. HIV-1 Subtyping by Phylogenetic Analysis

Firstly, a phylogenetic analysis was carried out to verify that the previously generated *pol* sequences clustered with those obtained from NFL sequencing. Then, the three data sets were separately analyzed by performing Mphy for HIV-1 subtype assignment. PR-RT, *pol*, and NFL sequences were first edited with BioEdit version 7.0.5.3 and aligned using Clustal W with full-length, most recently available, reference sequences of HIV-1 subtypes and CRFs retrieved from the HIV sequence databases (https://www.hiv.lanl.gov/content/sequence/NEWALIGN/align.html, accessed on 5 August 2025), using at least 4 reference sequences for each subtype/CRF, for a total of 840 sequences. Then, the complete alignments of PR-RT and *pol* sequences were manually trimmed from full-length genomes, and gaps were removed from the final alignments. Phylogenetic trees were constructed using the neighbor-joining method [45] by MEGA 12 [46], based on the Kimura 2-parameter (K2P) model [47]. The reliability of the branching orders was assessed by a bootstrap analysis of 1000 replicates [48]. To confirm subtype classification, a maximum likelihood tree with 1000 bootstrap replicates, a general time-reversible (GTR) nucleotide substitution model with gamma distribution among site heterogeneity, and a proportion of invariable sites (G + I + Γ) were inferred, and it was considered the best one by the MEGA 12 model test, as it showed the lowest Bayesian information criterion (BIC) score. A sequence that clustered monophyletically inside a clade with a bootstrap support value of ≥70.0% was assigned to that clade, as previously described [28]. The trees were rooted using midpoint rooting by FigTree software version 1.4.2 (https://tree.bio.ed.ac.uk/software/figtree/, accessed on 5 August 2025).

### 4.5. HIV-1 Subtyping by Automated Online Tools

Sequences from the three data sets were then separately analyzed by submitting them to the following automated subtyping tools: Stanford HIVdb v9.8 (https://hivdb.stanford.edu/hivdb/by-sequences/, accessed on 5 August 2025), COMET v2.4 (https://comet.lih.lu/, accessed on 5 August 2025), REGA v3.46 (http://dbpartners.stanford.edu:8080/RegaSubtyping/stanford-hiv/typingtool/, accessed on 5 August 2025), and Geno2pheno v3.5 (G2P) (https://subtyping.geno2pheno.org/, accessed on 5 August 2025). Regarding the reference sequence lists of the different tools, Stanford HIVdb v9.8’s list consists of 720 concatenated PR/RT/INT sequences, so that the comparison with NFL-based subtypes determined by Mphy could not be applicable. COMET v2.4’s list comprises 572 whole genome sequences [23], REGA v3.46’s comprises 87 whole genome sequences [20], and G2P considers in its database subtypes A1–A4, A6–A7, B, C, D, F1, F2, G, H, J, K, L, O, N, P, and only CRF01_AE as a recombinant form [22], so that other CRFs are shown as pure subtypes separated by commas.

### 4.6. Standardization of Subtyping Tool Assignments

Due to the fact that the outputs of automated tools are displayed differently, comparison between subtyping tools required standardization of the assignments. Thus, in COMET the terms “-check for” and “unassigned” were considered as disagreements in relation to Mphy results; in REGA the term “-like” was considered as the subtype or CRF identified by the tool, whereas the terms “recombinant of-” and “potential recombinant” were considered as discordant results in relation to Mphy, with the only exception of “recombinant of A1, G”, which was considered as CRF02_AG. Finally, in G2P, because of the lack of CRF reference sequences in its database, only the output “A1, G” was considered as a correctly identified recombinant (CRF02_AG), while other hypothetical recombinants were considered disagreements.

### 4.7. Concordance, Sensitivity, and Specificity

Performance of automated tools versus the gold standard Mphy was evaluated in terms of concordance (subtyping agreement), sensitivity (true positive rate), and specificity (true negative rate). Desirable target performance was set at ≥90.0%. As for concordance, when the automated tool assignment agreed with the one from Mphy, the result was considered concordant, otherwise it was considered discordant. Sensitivity and specificity were calculated using the formulas TP/(TP + FN) and TN/(TN + FP) [27], respectively, where TP is true positives, FP false positives, TN true negatives, and FN false negatives.

### 4.8. Statistical Analysis

To evaluate the presence of potential correlation between NFL sequencing success rate and the analyzed subtypes, Fisher’s exact test was used (https://astatsa.com/FisherTest/, 10 November 2025). A *p*-value < 0.05 was considered significant.

## Figures and Tables

**Table 1:** Reassigned HIV-1 subtypes performing Mphy on different portions of the genome.

	Mphy	
PR-RT	*pol*	NFL
B	B	CRF40_BF1
C	C	CRF31_BC
C	CRF08_BC	CRF64_BC
CRF02_AG	CRF06_cpx	CRF06_cpx
CRF02_AG	CRF02_AG	CRF63_02A6
CRF02_AG	CRF02_AG	CRF63_02A6
CRF12_BF	CRF12_BF	CRF44_BF1
CRF31_BC	CRF31_BC	CRF60_BC
F1	F1	CRF46_BF1
F1	F1	CRF46_BF1
F1	F1	CRF46_BF1
F1	F1	CRF46_BF1
G	CRF02_AG	CRF02_AG

The table reports the thirteen samples whose subtypes obtained by Mphy were reclassified when it was performed using NGS sequences of increasing length (PR-RT, *pol*, and NFL sequences). Reference sequences of HIV-1 subtypes and CRFs were retrieved from the HIV sequence databases (https://www.hiv.lanl.gov/content/sequence/NEWALIGN/align.html, accessed on 1 September 2025).

**Table 2:** Performance of HIV-1 subtyping tools in relation to Mphy conducted on PR-RT, *pol*, and NFL sequences in terms of sensitivity and specificity.

Subtype or CRF	N Mphy			% SENSITIVITY (95% CI ^a^)											
				Stanford v9.8			COMET v2.4			REGA v3.0			Geno2Pheno v3.5		
	PR-RT	*pol*	NFL	PR-RT	*pol*	NFL	PR-RT	*pol*	NFL	PR-RT	*pol*	NFL	PR-RT	*pol*	NFL
**B**	**14**	**14**	**13**	**100.0 (79.0–100.0)**	**100.0 (79.0–100.0)**		85.7 (63.5–92.5)	85.7 (63.5–92.5)	**92.3 (71.0–92.3)**	**92.9 (70.4–99.6)**	78.6 (57.6–78.6)	76.9 (54.6–76.9)	**92.9 (69.7–99.6)**	**92.9 (72.9–92.9)**	**100.0 (80.1–100.0)**
**A1**	**5**	**5**	**5**	**100.0 (55.2–100.0)**	**100.0 (55.2–100.0)**		**100.0 (49.1–100.0)**	80.0 (34.3–98.2)	80.0 (36.1–80.0)	**100.0 (48.9–100.0)**	80.0 (31.5–98.9)	**100.0 (48.9–100.0)**	**100.0 (55.2–100.0)**	80.0 (36.1–80.0)	80.0 (36.1–80.0)
**A6**	**7**	**7**	**7**	**100.0 (65.7–100.0)**	**100.0 (65.7–100.0)**		**100.0 (65.7–100.0)**	**100.0 (65.7–100.0)**	**100.0 (65.7–100.0)**	**NA**			**100.0 (65.7–100.0)**	**100.0 (65.7–100.0)**	0.0 (0.0–0.0)
**C**	**7**	**6**	**5**	**100.0 (63.5–100.0)**	**100.0 (57.5–100.0)**		**100.0 (63.5–100.0)**	**100.0 (57.5–100.0)**	**100.0(51.5–100.0)**	85.7 (50.4–85.7)	**100.0 (57.5–100.0)**	**100.0 (51.5–100.0)**	**100.0 (63.5–100.0)**	**100.0 (57.5–100.0)**	**100.0 (50.7–100.0)**
**G**	**5**	**4**	**4**	80.0 (36.1–80.0)	**100.0 (43.9–100.0)**	**NA**	80.0 (36.1–80.0)	**100.0 (47.3–100.0)**	25.0 (1.4–25.0)	80.0 (36.1–80.0)	**100.0 (47.3–100.0)**	75.0 (26.2–75.0)	80.0 (33.3–98.9)	**100.0 (43.2–100.0)**	**100.0 (47.3–100.0)**
**F1**	**13**	**13**	**9**	**100.0 (80.1–100.0)**	**100.0 (80.1–100.0)**		84.6 (62.6–84.6)	61.5 (39.5–61.5)	55.6 (27.8–55.6)	**92.3 (71.0–92.3)**	**100.0 (80.1–100.0)**	88.9(54.2–99.4)	**92.3 (71.0–92.3)**	84.6 (62.6–84.6)	44.4 (18.8–44.4)
**CRF01_AE**	**7**	**7**	**7**	**100.0 (65.7–100.0)**	**100.0 (65.7–100.0)**		**100.0 (65.7–100.0)**	**100.0 (65.7–100.0)**	85.7 (50.4–85.7)	**100.0 (65.7–100.0)**	**100.0 (65.7–100.0)**	**100.0 (65.7–100.0)**	**100.0 (65.7–100.0)**	**100.0 (65.7–100.0)**	**100.0 (65.7–100.0)**
**CRF02_AG**	**16**	**16**	**14**	**100.0 (82.3–100.0)**	**100.0 (82.3–100.0)**		50.0 (30.4–55.9)	62.5 (43.5–62.5)	64.3 (43.3–64.3)	6.3 (0.3–12.2)	18.8 (6.0–18.8)	28.6 (11.2–35.3)	43.8 (24.8–49.7)	75.0 (56.0–75.0)	28.6 (11.2–35.3)
**CRF12_BF**	**9**	**9**	**8**	55.6 (27.8–55.6)	55.6 (27.8–55.6)		55.6 (27.8–55.6)	33.3 (10.9–33.3)	0.0 (0.0–0.0)	11.1 (0.6–11.1)	0.0 (0.0–0.0)	0.0 (0.0–0.0)	**NA**		
															
**Subtype or CRF**	**N Mphy**			**% SPECIFICITY (95% CI ^a^)**											
				**Stanford v9.8**			**COMET v2.4**			**REGA v3.0**			**Geno2Pheno v3.5**		
	**PR-RT**	***pol***	**NFL**	**PR-RT**	***pol***	**NFL**	**PR-RT**	***pol***	**NFL**	**PR-RT**	***pol***	**NFL**	**PR-RT**	***pol***	**NFL**
**B**	**14**	**14**	**13**	**97.5 (93.8–97.5)**	**98.7 (95.2–98.7)**		**98.7 (94.8–99.9)**	**98.7 (94.8–99.9)**	**100.0 (96.5–100.0)**	**97.5 (93.5–98.7)**	**100.0 (96.3–100.0)**	**100.0 (96.4–100.0)**	**96.2 (92.1–97.4)**	**100.0 (96.5–100.0)**	**100.0 (96.8–100.0)**
**A1**	**5**	**5**	**5**	**100.0 (97.5–100.0)**	**100.0 (97.5–100.0)**		**92.0 (89.2–92.0)**	**98.9 (96.3–99.9)**	**100.0 (97.5–100.0)**	**90.9 (88.0–90.9)**	**90.9 (88.2–92.0)**	**90.9 (88.0–90.9)**	**100.0 (97.5–100.0)**	**100.0 (97.5–100.0)**	**100.0 (97.5–100.0)**
**A6**	**7**	**7**	**7**	**100.0 (97.2–100.0)**	**100.0 (97.2–100.0)**		**100.0 (97.2–100.0)**	**100.0 (97.2–100.0)**	**100.0 (97.2–100.0)**	**NA**			**100.0 (97.2–100.0)**	**100.0 (97.2–100.0)**	**100.0 (100.0–100.0)**
**C**	**7**	**6**	**5**	**98.8 (95.9–98.8)**	**97.7 (94.8–97.7)**		**98.8 (95.9–98.8)**	**97.7 (94.8–97.7)**	**97.7 (95.0–97.7)**	**100.0 (97.1–100.0)**	**97.7 (94.8–97.7)**	**97.7 (95.0–97.7)**	**98.8 (95.9–98.8)**	**97.7 (94.8–97.7)**	**96.6 (93.8–96.6)**
**G**	**5**	**4**	**4**	**100.0 (97.5–100.0)**	**97.8 (95.2–97.8)**	**NA**	**100.0 (97.5–100.0)**	**100.0 (97.6–100.0)**	**100.0 (98.9–100.0)**	**100.0 (97.5–100.0)**	**100.0 (97.6–100.0)**	**100.0 (97.8–100.0)**	**97.7 (95.1–98.8)**	**96.6 (94.1–96.6)**	**100.0 (97.6–100.0)**
**F1**	**13**	**13**	**9**	**100.0 (96.8–100.0)**	**100.0 (96.8–100.0)**		**100.0 (96.4–100.0)**	**100.0 (96.4–100.0)**	**100.0 (97.0–100.0)**	**100.0 (96.5–100.0)**	**100.0 (96.8–100.0)**	**91.7 (88.0–92.8)**	**100.0 (96.5–100.0)**	**100.0 (96.4–100.0)**	**100.0 (97.3–100.0)**
**CRF01_AE**	**7**	**7**	**7**	**100.0 (97.2–100.0)**	**100.0 (97.2–100.0)**		**100.0 (97.2–100.0)**	**100.0 (97.2–100.0)**	**100.0 (97.1–100.0)**	**100.0 (97.2–100.0)**	**100.0 (97.2–100.0)**	**100.0 (97.2–100.0)**	**100.0 (97.2–100.0)**	**100.0 (97.2–100.0)**	**100.0 (97.2–100.0)**
**CRF02_AG**	**16**	**16**	**14**	**98.7 (95.0–98.7)**	**98.7 (95.0–98.7)**		**98.7 (94.6–99.9)**	**100.0 (96.1–100.0)**	**100.0 (96.3–100.0)**	**98.7 (97.5–99.9)**	**100.0 (97.3–100.0)**	**98.7 (95.6–99.9)**	**98.7 (94.8–99.9)**	**100.0 (96.1–100.0)**	**98.7 (95.6–99.9)**
**CRF12_BF**	**9**	**9**	**8**	**100.0 (97.0–100.0)**	**100.0 (97.0–100.0)**		**100.0 (97.0–100.0)**	**100.0 (97.6–100.0)**	**100.0 (100.0–100.0)**	**100.0 (98.9–100.0)**	**100.0 (100.0–100.0)**	**100.0 (100.0–100.0)**	**NA**		

The table reports sensitivity and specificity of subtypes present in at least 3 sequences analyzed. The values with automated tool performances of >90% are shown in bold. The not-applicable (NA) values indicate the absence of the reference sequences in the automated tool algorithm or the lack of whole genome reference sequences in the tool. ^a^ CI, confidence interval. Reference sequences of HIV-1 subtypes and CRFs were retrieved from the HIV sequence databases (https://www.hiv.lanl.gov/content/sequence/NEWALIGN/align.html, accessed on 1 September 2025).

## Data Availability

Data are contained within the article.

## Supplementary Materials

The following supporting information can be downloaded at: https://www.mdpi.com/article/10.3390/ijms262311666/s1.
