# Supplementary material for: Improved HIV-1 Subtyping Accuracy Using near Full-Length Sequencing: A Comparison of Common Tools
Source: Int J Mol Sci. 2025 Dec 2;26(23):11666. doi: 10.3390/ijms262311666 (PMC12691708; doi:10.3390/ijms262311666)
Supplement: Supplementary file 1 [file ijms-26-11666-s001.zip › ijms-4010157-supplementary.pdf]

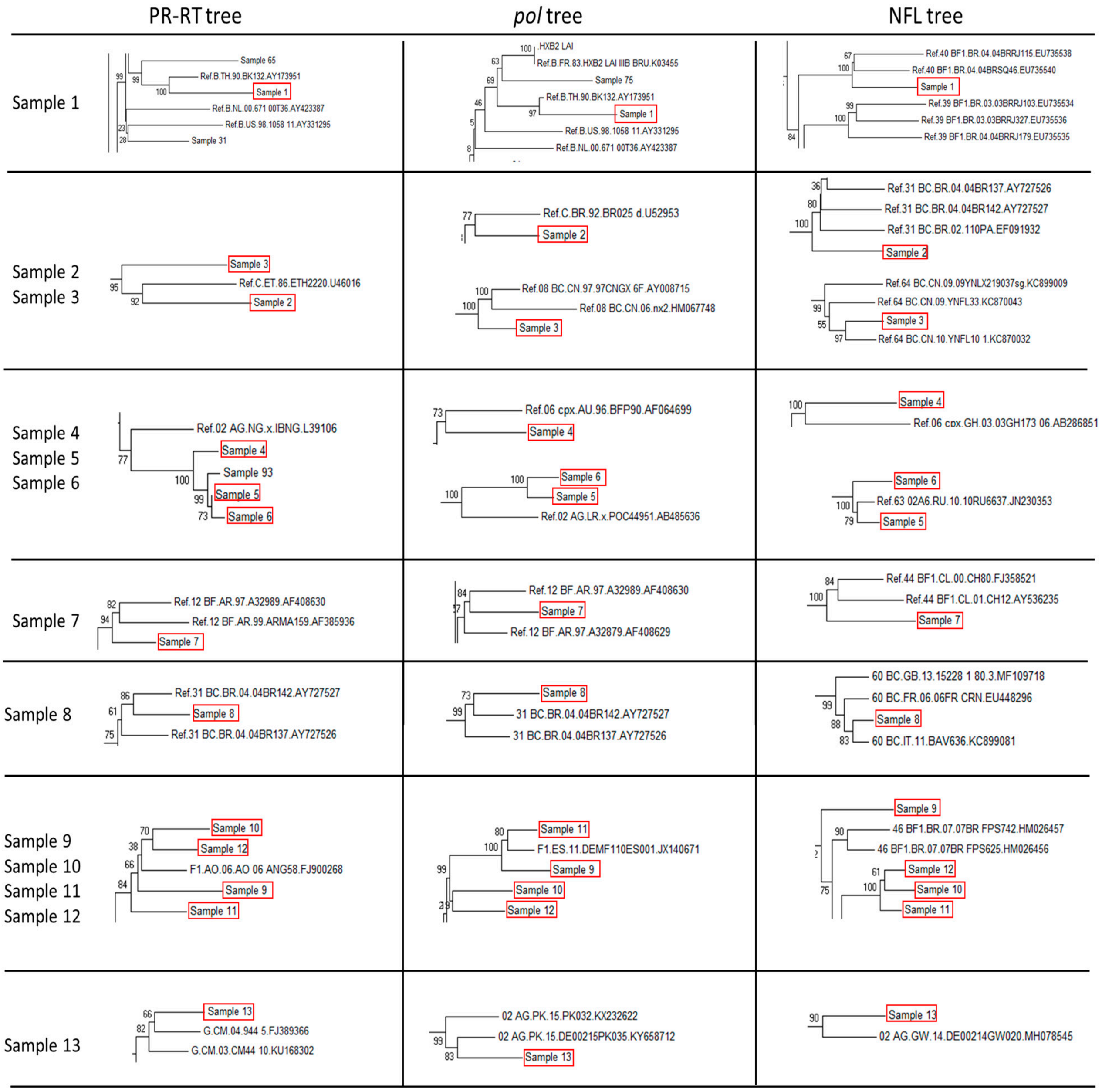

Supplementary Figure S1. Maximum likelihood phylogenetic trees details representing the 13 samples whose subtypes obtained by Mphy were reclassified when it was performed using NGS sequences of increasing length (PR-RT, *pol* and NFL sequences). Reference sequences of HIV-1 subtypes and CRFs were retrieved from the HIV sequence databases (https://www.hiv.lanl.gov/content/sequence/NEWALIGN/align.html, accessed on 1 September 2025).
